# Analyzing Exercise Behaviors during the College Years: Results from Latent Growth Curve Analysis

**DOI:** 10.1371/journal.pone.0154377

**Published:** 2016-04-28

**Authors:** Jean Lemoyne, Pierre Valois, Werner Wittman

**Affiliations:** 1 Department of Human Kinetics, Université du Québec à Trois-Rivières, Trois-Rivières, Canada; 2 Faculty of Education, Université Laval, Quebec City, Canada; 3 Department of Psychology, University of Mannheim, Mannheim, Germany; Tokyo Institute of Technology, JAPAN

## Abstract

The objective of this study is to analyze changes in the predictors of physical activity behavior among college students. The Theory of Planned Behavior served as its theoretical framework. Methods: Among an initial sample of 417 college students, 195 participants completed a validated questionnaire measuring attitudes, subjective norms, perceived control, intentions and self-reported physical activity, at the beginning and end of each of 3 college semesters. Latent growth curve modeling analyses were conducted to examine the relationships between the trajectories of changes in PA, intentions, attitudes, subjective norms, and perceived control. Results: Good fit indices supported the validity of the proposed longitudinal model (CFI > .97, RMSEA < .05). Changes in perceived control (γ = 0.57) were significantly linked with changes in intentions (*p* < .05). Perceived control (γ = 0.28) and intention growth (γ = 0.36) predicted behavior changes (*p* < .05). No gender differences were observed on attitudes, subjective norms and perceived control (*p* > .10). However, girls tend to have higher growth parameters on intentions and physical activity (*p* < .05). In summary, intentions and physical activity has significantly increased over 3 college semesters (growth parameters significant at *p* < .05). Conclusions: This study demonstrated that attitudes and perceived control are key determinants regarding the intentions of being active. On a longer term perspective, future physical activity interventions should focus on the enhancement of students’ perceived control. Such educational context should help in promoting the adoption of an active lifestyle during college.

## Introduction

Late adolescence seems to be the stage with the steepest decline in physical activity (PA) behaviors [1, 2]. A meta-analysis disclosed that PA behaviors diminish during college [3]. Nelson and colleagues [4] reported significant unfavorable shifts in the level of PA, especially among late adolescent girls, with more than 50% of them being physically inactive compared to 40% of boys. Thus, a better understanding of the mechanisms explaining adoption and maintenance of PA in late adolescence is especially relevant.

**Fig 1:**
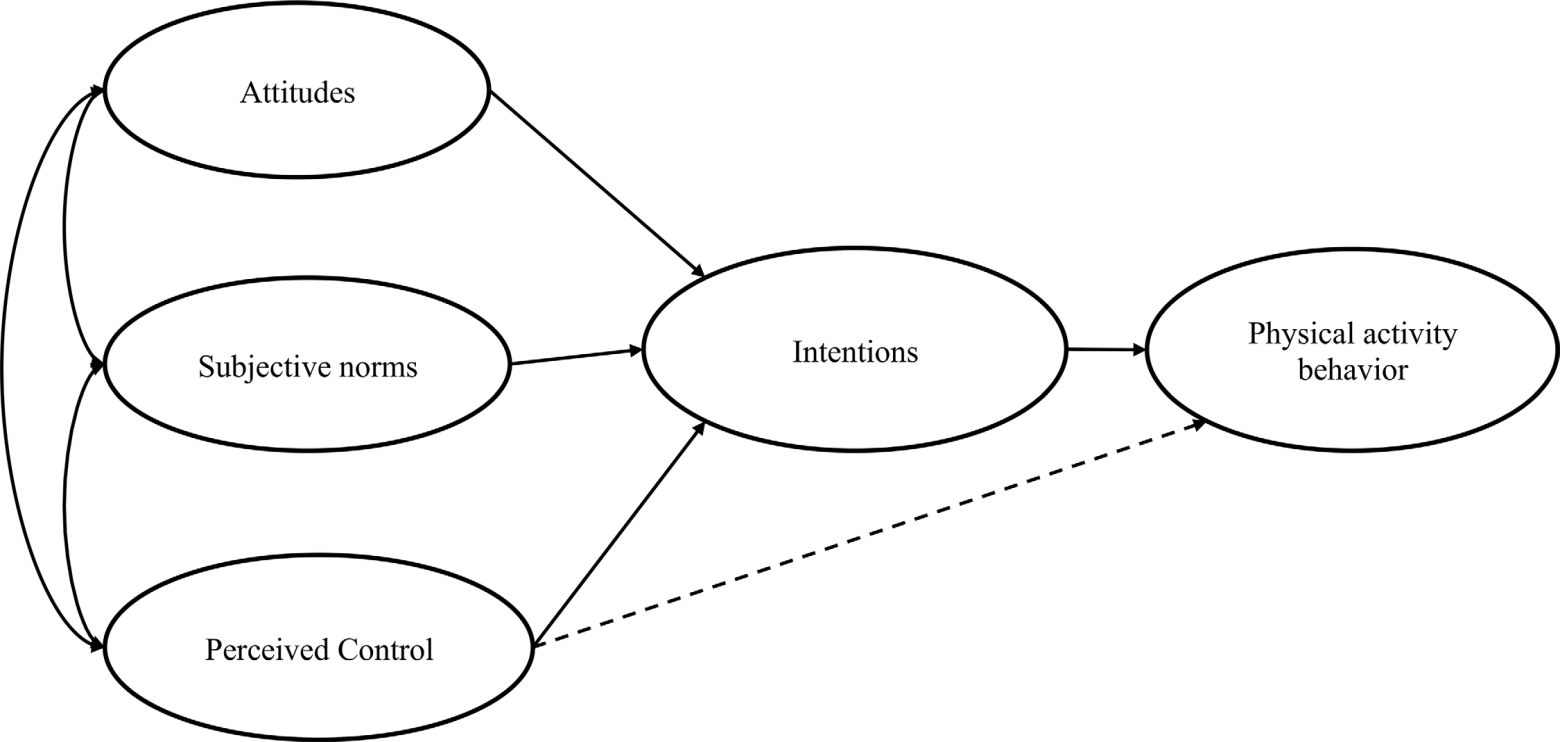
The theory of planned behavior (Ajzen, 2012).

An extensive amount of work has been conducted in the past to shed light on this trend. Among the plethora of behavioral theories related to exercise, the theory of planned behavior (TPB) figures as one of the most frequently cited [5]. The TPB (Fig 1) is a useful theoretical model, especially because it allows stakeholders to design educational interventions based on a behavior’s determinants. TPB assumes that a specific behavior is determined by intentions towards this behavior. Intentions, for their part, are shaped by 3 factors: attitudes (A), subjective norms (SN), and perceived behavioral control (PBC). Attitudes (A) reflect an individual’s beliefs about the affective and instrumental attributes of a behavior. An individual who perceives regular PA as fun and important for health is more inclined to develop strong intentions towards exercise. SN reflects the motivation of an individual to act as it is encouraged by his environment (peers, parents). Finally, PBC reflects an individual’s perceptions about his capabilities towards a behavior.

Ajzen [6] specified that control beliefs over external factors, such as perceived barriers (lack of time, motivation) and facilitators (accessibility to installations, abilities) could influence intentions about PA. From this perspective, an individual who is confident about his capabilities towards a behavior should be more inclined towards intending to perform the behavior. In this regard, Ajzen [7] suggested the possibility of a direct path involving PBC and behavior. This hypothesis was supported by Sniehotta et al [8], who demonstrated that PBC was directly related to exercise participation in a sample of 307 adults, following an intervention study.

Empirical evidence suggests that, from childhood to young adulthood, boys are more active than girls [4]. Moreover, there seem to be gender differences in TPB variables. Trinh, Rhodes & Shon [9] reported that the relationship between PBC and intentions about PA one month later was stronger among boys, comparatively with girls. However, in this study, despite an increase for boys’ PBC, relationships involving A, SN and intentions were similar regarding gender. Another cross sectional study, involving 621 college students showed that males’ PA was explained only by intentions, whereas females’ PA was dictated by intentions, self-efficacy, and attitudes [10]. Such results indicate the possible presence of gender differences regarding the relative contribution of each of the TPB on PA.

TPB assumes that changes in its proximal factors should determine changes in intentions towards PA. Consequently, changes in intentions should predict changes in behavior. Empirical data support the plausibility of the “changes predict changes” hypothesis. Digelidis et al. [11] demonstrated that changes in attitudes were significantly linked with changes in exercise participation in a cohort of 260 adolescents. Webb and Sheeran [12] ascertained that growth in intentions was associated with increased exercise behaviors. However, Lippke and Ziegelman [13] criticized the fact that behavior change was studied in an oversimplified static way, by measuring individual differences in predictors at baseline, and using these measures to predict differences in behavior at follow-up. To counteract this limitation, emerging methods of analysis have been developed and are now used regularly [14]. Dynamic statistical approaches, such as latent growth modelling (LGM), allow researchers to counteract these limitations, especially in the quest to refine our understanding of behavior change. LGM analyses changes over time, and is conducted in longitudinal designs of 3 or more waves of assessment. However, especially in the PA domain, few studies have investigated behavior change processes with these emerging methods.

By employing LGM, Sniehotta and colleagues [15] demonstrated that over 6-week follow-up, changes in intentions predicted changes in PA among an adult sample. More recently, Reuter et al. [16] conducted a longitudinal study among a sample of 850 adults, and demonstrated that changes in self-efficacy and intentions predicted changes in exercise. However, despite obtaining promising results, such studies need to be conducted among other populations, to refine our understanding of the "changes predict changes" hypothesis. Studies need to be replicated among younger populations, such as in late adolescence, with longer follow-ups. Therefore, the present investigation aims to test the "changes predict changes" hypothesis among a cohort of college students.

## Methods

### The present study: analyzing behavior change among college students

In Quebec (Canada), college is a stage between high school and university that aims to prepare students for university studies. Physical education (PE) is mandatory in Quebec colleges. The Quebec College PE program consists of 3 semesters of 15-week courses of 2 hours each. Throughout this program, semesters are oriented towards multiple objectives, such as: knowledge about PA and awareness towards health behaviors. Such context offers an excellent opportunity to verify the evolution of PA correlates during college.

The present study aims to test the TPB in a longitudinal context, by exploring the “changes predict changes” hypothesis during 3 college semesters. Specifically, this study has 3 objectives. First, it will verify if growth parameters of TPB variables predict changes in students’ intentions during participation in PE. Second, this investigation verifies if PA changes are explained by the TPB constructs. Finally, this study will verify if the growth parameters of A, SN, PBC, intentions, and PA evolve differently regarding gender.

### Participants and design

From the fall of 2008, students enrolled in their first PE classes were asked to participate. Those who accepted completed a consent form. Permission to conduct the study was granted by the Laval University Ethics for Research Committee (2008-211- R1-06-08-2009). According to the Ethical Committee (ERC): *A minor of 14 years and more can consent only to research if*, *in the opinion of one EC designated*, *it has only minimal risk to his health that circumstances of research warrant*. Regarding this, no consent forms from the parents were required because all participants were 16 years old or higher. Participants provided signed consent forms and completed the questionnaires, returning them to the research assistant. Same procedures were undertaken at the beginning and end of each of 3 semesters, resulting in 6 waves of assessment over a 21-month period. At T1, 417 students (271 females– 68%; 146 males– 32%) were enrolled in PE classes. These proportions are representative of Quebec’s college population [17]. At the end, 195 students (17.8 ± 2.4 years) completed the six waves of assessment. Multiple causes could explain attrition: withdrawal from college, changes in academic paths, and, lack of interest in the study. To verify if the pattern of missing data could be specific to participants, we conducted independent *t* tests on each of the TPB variables at T1, between those who did not take part in the full design (*n* = 222), and the final sample (n = 195). For each variable initial scores (A, SN, PBC, I, and PA), no mean differences were observed between the sub-samples (all at *p*>.05).

### Measures

**Gender** was categorized in 2 scores (female = 1, male = 2). Thus, higher values in intercept factors would mean higher initial values for boys. For slope factors, positive values would mean a higher rate of change for boys, whereas negative values would mean higher growth rate for girls.

**Physical activity** was defined as suggested by the Canadian Society for Exercise Physiology [18], i.e., taking part in 3 or more weekly sessions of moderate to vigorous PA. PA was measured with 2 items. First, we asked participants about the frequency of participation in moderate to vigorous PA. Participants had to answer “Over the last 3 months, how many days per week (0 to 7) did you take part in sweat-inducing PA, such as jogging or participating in sports”. This single measure of PA was considered in earlier research and revealed good reliability, when correlated with objective measures [19]. Second, participants had to rate their level of participation in PA over the last 3 months on a 5-point Likert type scale, ranging from 1 (sedentary) to 5 (seriously involved in PA). Mean and median correlations between the 2 items at each time of assessment were high (*r*_*mean*_ varying between .55 and .72, and *r*_*median*_ varying between .65 and .68). We calculated composite scores for PA behavior, by calculating the mean score of these two items. In the present investigation, because TPB assumes that TPB constructs are predictors of further PA, we take account of five PA scores among the six measures (see analyses for more details).

**Intentions** about participating in regular PA were measured with a 7-point Likert type scale consisting of 3 items scoring from -3 (totally disagree) to +3 (totally agree): “For the next 3 months, I intend (item 1), will try (item 2), and will plan (item 3) to get involved in regular PA”. Preliminary analysis with the intentions scale revealed very good reliability (α >.92). For each assessment, six composite (item parceling) intention scores were analyzed.

**Attitudes (A)** were measured with a semantic scale based on 7 items. First, participants were asked to complete the sentence: “For me, taking part in regular PA is…", and had to rate their responses with 6-point bipolar adjectives: fun-dull, useful-useless, healthy-unhealthy, beneficial-harmful, desirable-undesirable, motivating-discouraging, and pleasant-unpleasant. Preliminary analysis revealed very good reliability of this scale (α = .86). For each assessment, item parceling composite scores were calculated for analyses, resulting in six composite attitude scores.

**Subjective Norms (SN)** were measured with 4 items from a Likert-type 7-point scale (1, not motivated at all to 7, extremely motivated) about participants’ motivation to comply with the opinion of people from their social environment. They were asked: “For the next 3 months, how motivated will you be to act as your (1) parents (2) peers, (3) doctor, and (4) physical education teacher, if they recommend that you to take part in regular PA”. Preliminary analyses revealed acceptable reliability for the scale (α = .71). Similarly to A, SN were measured with composite scores, resulting in six SN composite scores.

**Perceived control (PBC)** was measured with a 15-item multiplicative “expectancy-value” sub-scale. The scale contained 8 perceived barriers and 7 perceived facilitating factors for PA. Participants had to rate the likelihood of occurrence of these factors on a 7-point scale (from -3, *do not agree at all*, to +3, *totally agree*) (i.e. “Over the next 3 months, I think that the following situations will occur”). The power of these factors was also measured on a 7-point Likert scale ranging from 1 (*no*, *not at all probable*) to 7 (*extremely probable*) (i.e. “During the next year, the following situations will help me to adopt regular PA”). For each pair of items, scores ranged between -21 and +21. Perceived barrier scores were reversed, because highly perceived barriers reflect unfavorable beliefs. Preliminary analyses revealed acceptable reliability of this scale (α = .72). Composite scores were calculated by obtaining mean scores for all items. Same procedure was repeated at each wave of assessment, resulting in six PBC scores. Positive scores with this scale meant favorable PBC towards regular PA.

### Statistical analyses

**Fig 2:**
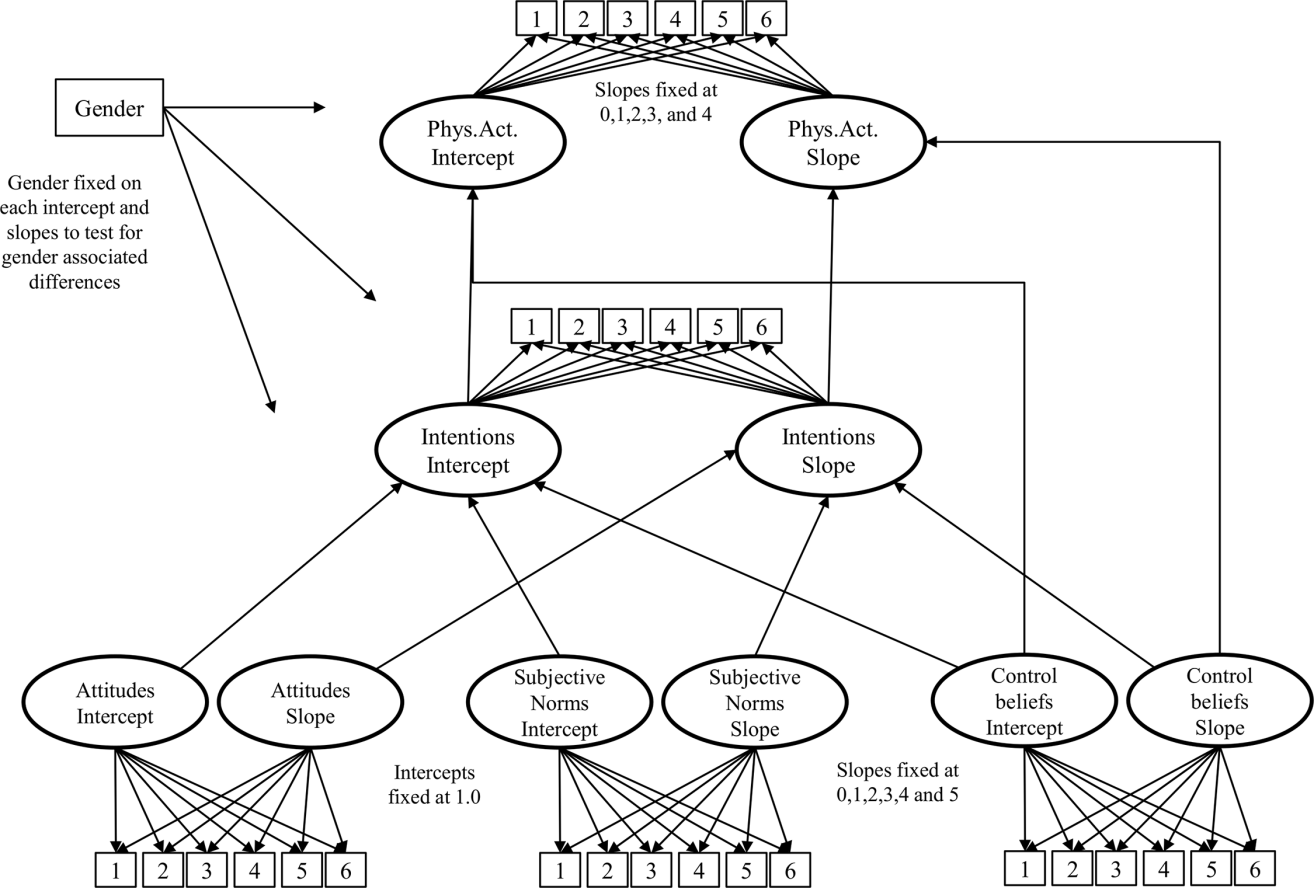
Latent growth model for the present study. Solid lines represent relationships between intercept factors; dotted lines represent relationships between slope factors.

Latent growth curve (LGC) modeling was undertaken to test the longitudinal TPB model (Fig 2). Model identification followed the recommendations of Little [20]. LGC modeling estimates 2 parameters: 1) the intercept, and 2) the slope. The intercept reflects initial values for each of the model’s constructs, that is, A, SN, PBC, intentions, and behavior. The slope represents the rate of linear growth for each construct, that is, through the entire repeated measures design.

For A, SN, PBC, and intentions, intercept factors were fixed at a value of 1.0 at the first semester. Slope factors for these constructs estimated linear change, as loading for slope factors was fixed at values of 0, 1, 2, 3, 4, and 5. For physical activity behavior, the intercept was fixed at the end of the first semester (Time 2) and was re-centered (0, 1, 2, 3, 4), with 5 self-reported PA measures. In this manner, fixing the PA intercept at Time 2 estimated paths at initial status, and predicted behavior with the intercepts of TPB model variables, because their initial values were measured at a preceding wave. A positive growth parameter would mean an increase in PA over 3 semesters. As assumed by the TPB, correlations between the intercept factors for each of the predictors (A, SN, PBC) were included in the model. Covariance coefficients between slope factors of each TPB distal variable were added to the model, to verify if baseline measures of A, SN and PBC were related with their growth parameters. Finally, gender was added as a predictor on the intercept and slope factors to verify gender-associated differences regarding slopes in each variable.

### Missing data and model estimation

Missing data are common in longitudinal studies and should be treated cautiously. To reduce the possibility of biased estimates, we undertook full information maximum likelihood (FIML) estimation, as it is recommended [21, 22]. When data deviate from normality, the model is fitted with robust estimators. Similar analyses were performed in earlier studies and gave reliable estimates [23].

Analyses were conducted with EQS software (see Little [20] for further explanation). In LGC, the chi-square statistic indicates data fit to the proposed model. However, it has been shown that chi-square statistic is too sensitive to size sample, resulting in interpretation bias [24]. Thus, we used other fit indices to estimate the proposed model. The comparative and non-normed fit indices (CFI & NNFI) are indicative of relationships between parameters in the model. For these indices, values over .90 suggest very good adjustment [24]. The rooted mean square error of approximation (RMSEA) gives an approximation of data adjustment. RMSEA with values below .08 is considered as a satisfactory adjustment index [25]. As *post hoc* analyses, we employed the Wald (drop parameters) and the Lagrange (add parameters) tests.

## Results

### Sample size and descriptive statistics

Descriptive statistics (mean scores for each wave of assessment) are reported in Table 1. Table 2 shows correlations between the model’s constructs at Time 1 are reported in Table 1. PA was correlated with intentions (r = .57, *p* < .001), A (.20, *p* < .01), SN (.09, *p* < .05), and PBC (r = .34, *p* < .05). Intentions were correlated with TPB variables (r_A-INT_ = .31, r_SN-INT_ = .13, and r_CB-INT_ = .33, all at *p* < .05).

**Table 1:** Descriptive statistics at each wave of assessment.

Time of assessment	PA (M±SD)	INT (M±SD)	A (M±SD)	SN (M±SD)	PBC (M±SD)
Time 1	2.45 ± 1.7	1.52 ± 1.5	4.62 ± 0.7	5.28 ± 1.2	4.58 ± 2.9
Time 2	2.41 ± 1.4	1.24 ± 1.5	4.80 ± 0.8	5.11 ± 1.2	3.89 ± 3.2
Time 3	2.39 ± 1.5	1.52 ± 1.4	4.83 ± 0.8	5.18 ± 1.1	3.24 ± 3.6
Time 4	2.58 ± 1.6	1.62 ± 1.3	4.84 ± 0.7	5.05 ± 1.2	3.31 ± 3.3
Time 5	2.47 ± 1.9	1.78 ± 1.2	4.79 ± 0.7	5.31 ± 1.0	3.86 ± 3.4
Time 6	3.54 ± 1.2	1.57 ± 1.5	4.82 ± 0.8	5.18 ± 1.1	2.91± 3.3

PA: physical activity, INT: intentions, A: attitudes, SN: subjective norms, PBC: perceived control.

**Table 2:** Inter-correlations between constructs at the first wave of assessment. Bold characters represent inter-correlations of the same constructs between each wave of assessment (Time 1 to Time 6).

Variables	PA	INT	A	SN	PBC
PA	0.83***				
INT	0.57***	0.88***			
A	0.20**	0.31**	0.80***		
SN	0.09*	0.13**	0.21**	0.86***	
PBC	0.34**	0.38**	0.37**	0.10*	0.80***

PA: physical activity, INT: intentions, A: attitudes, SN: subjective norms, PBC: perceived control.

*p < .05

**p < .01

***p < .001.

As shown on Table 2, TPB variables were significantly correlated with each other (r_A-SN_ = .21, r_SN-PBC_ = .17, and r_PBC-A_ = .24, all at *p* < .01). Reliability scores revealed good stability for each construct over the six waves of assessment (Cronbach’s αs across six assessments, varying from .80 to .88).

### Goodness of fit for the longitudinal TPB model

Due to deviation from normality (for PA measures, we decided to base our interpretations on FIML robust fit indices. Results revealed acceptable fit indices, meaning good data adjustment to the model: *χ*² = 827.78 (*df* = 436), *p* < .001, NNFI = .977, CFI = .979, RMSEA = .025. Table 2 discloses unstandardized equations for intercepts and slopes. Mean intercept scores revealed information about the average scores for each construct. However, these scores were not useful to test the TPB longitudinal model. When we tested the TPB model with the intercept of each factor (Table 3, right), attitudes (γ = 0.16, *p* < .05) and PBC (γ = 0.20, *p* < .05) significantly predicted participants’ intentions to take part in regular PA, but not SN (γ = 0.06, *p* > .05). Coherently with TPB assumptions, intentions (γ = 0.54, *p* < .05) predicted self-reported PA at the end of the first semester (Time 2). PBC was not significantly associated with PA intercept (γ = 0.01, *p*>.05). We also analyzed growth parameters of each TPB variable. Over 3 PE semesters, attitudes (π = 0.82, *p* < .05), intentions (π = 0.06, *p* < .05), and PA (π = 0.12, *p* < .05) increased significantly. However, PBC decreased (π = -0.27, *p* < .05). No significant differences were observed on SN growth parameter (π = -.02, *p*>.05).

**Table 3:** Construct equations (unstandardized parameters) for initial status and growth parameters of each of the model’s constructs.

	Initial status		Linear change		Predicting initial status		Changes predict changes	
	Mean score	Gender coefficient	Average growth	Gender coefficient	INT	PA	INT	PA
**PA**	2.72	0.65	0.12	-0.10	N3T	N/T	N/T	N/T
**INT**	1.37	0.55	0.06	-0.10	N/T	0.54	N/T	0.36
**A**	3.86	n.s.	0.82	n.s.	0.16	n.s.	n.s.	n.s.
**SN**	5.21	n.s.	n.s.	n.s.	n.s.	N/T	n.s.	N/T
**PBC**	4.37	n.s.	-0.27	n.s.	0.20	n.s.	0.57	

PA: physical activity, INT: intentions, A: attitudes, SN: subjective norms, PBC: perceived control.

All parameters are significant at *p* < .05, n.s.: non-significant (*p*>.05), N/T: not tested.

The first objective of this study was to verify the “changes predict changes” part of the model, by testing paths between the constructs’ slopes, and attesting if changes observed in the TPB constructs predicted changes in intentions, and if intentions and PBC changes predicted changes in PA. Analyses revealed that changes in students’ intentions to take part in regular PA were explained by changes in PBC (γ = 0.57, *p* < .05), but not by changes in attitudes (γ = 0.07, *p*>.05), and SN (γ = -0.09, *p*>.05). The second objective of this study was to verify the prediction of linear change in self-reported PA. Changes in PA were explained by intentions (γ = 0.28) and PBC (γ = 0.36, *p* < .05), suggesting that the decrease in PBC was associated with smaller changes in PA.

**Fig 3:**
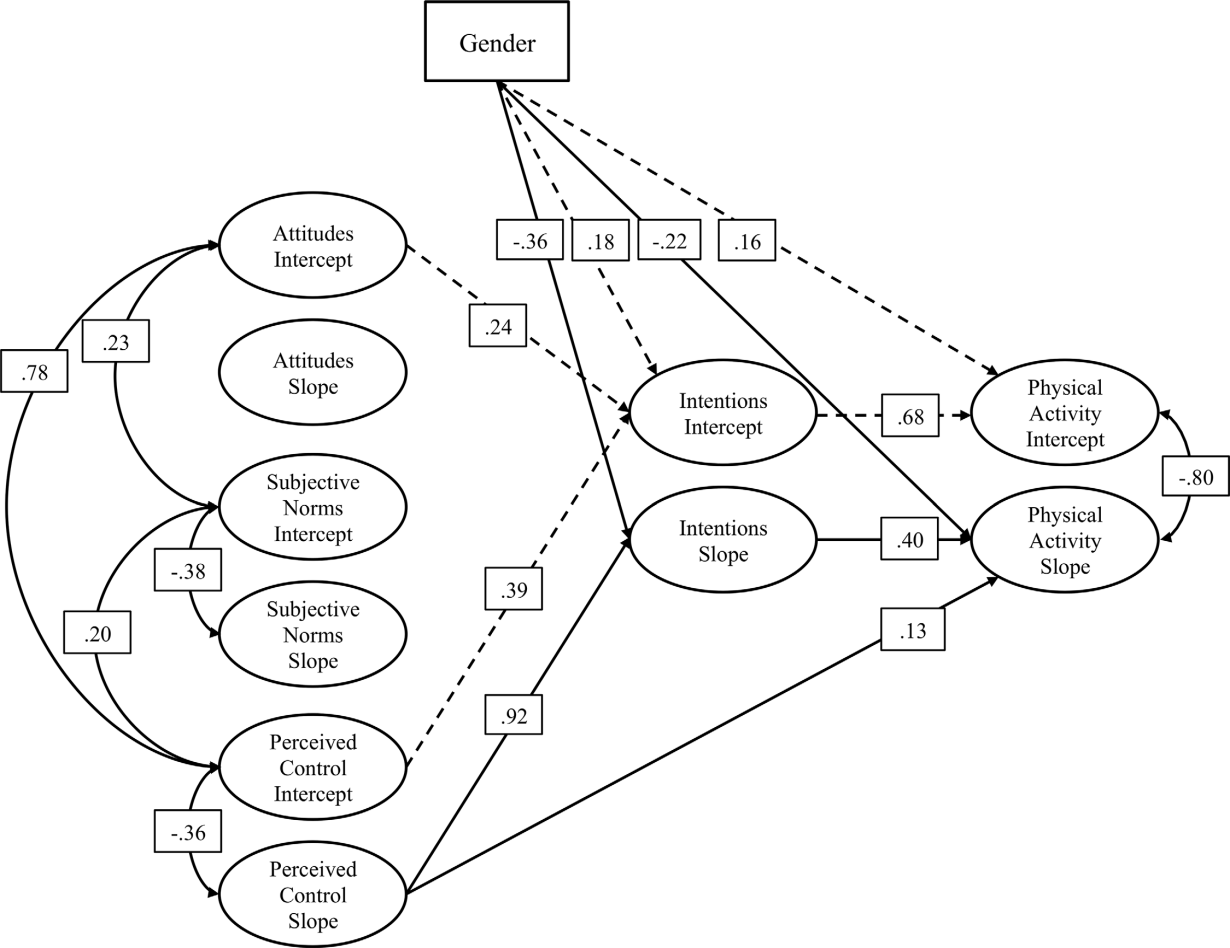
Standardized solution: the TPB longitudinal model (All illustrated paths are significant at *p* < .05).

The third objective of this study was to verify the presence of gender-associated differences in the longitudinal TPB model. At Time 1 (Table 2), baseline measures revealed no gender-associated differences on initial scores for A (τ = -0.28), SN (τ = 0.07), and PBC (τ = 0.43). However, boys had higher intentions at T1 (τ = 0.55, *p* > .05). At the end of the first semester (Time 2), PA was also higher among boys (τ = 0.65, *p*>.05). Growth parameter analyses revealed that changes in intentions (τ = -0.12, *p*>.05) and behavior (τ = -0.10, *p*>.05) were significantly higher among girls. Covariance between each construct’s intercept were calculated, revealing significant relationships between A and SN (ϕ = 0.23, *p* < .05), and between SN and PBC (ϕ = 0.20, *p* < .05). Fig 3 represents the standardized solution for the TPB longitudinal model. Covariance coefficients were also calculated between each intercept and its corresponding slope. As illustrated, significant negative covariance coefficients were obtained for: SN (ϕ = -0.38, *p* < .05), and PBC (ϕ = -0.36, *p*>.05). This suggests that participants who had lower initial values on A and SN showed a higher rate of change on these variables over the 3 semesters.

## Discussion

The main purpose of this study was to test the TPB structure in a longitudinal design. In summary, we tested paths relating growth parameters for each of the TPB constructs, and their relationships with intentions and PA changes. Finally, we tested for gender-associated differences in the proposed model. To our knowledge, it was the first study to take account of the TPB model with a 2-year, 6-wave data collection design. Our results showed that during the college years, there were significant increases in students’ physical activity behaviors. This increase could be attributable to the fact that physical education classes are mandatory, resulting in a possible increase of PA behaviors. However, due to the lack of a control group, the present study cannot confirm that these outcomes are essentially due to the quality of physical education program. In the province of Quebec, physical education is mandatory, and the increase in PA could be attributable to the fact that students had to participate in physical education to obtain their college diplomas. Despite having modest influence on the level of PA, other positive outcomes are coupled with physical education [26]. That is why we also emphasized the evolution of other variables than PA only as other potential outcomes of physical education.

From this perspective, our results revealed small, but significant changes on the participants’ attitudes. Inversely no significant changes were observed on subjective norms. According to Fishbein and Ajzen [27], it is plausible to suggest that past experiences related with physical education could contributed to stabilize attitudes and subjective norms towards physical activity, resulting in non-significant changes. However, at initial status, significant relationships between the participants’ attitudes with self-reported physical activity at Time 1 shows that attitudes reflect an individual’s predisposition towards behavior (stronger intentions). However, no significant relationships were observed between attitudes changes and intention changes. In this regard, participants’ antecedent experiences in physical education may have contribute to a possible crystallization of attitudes, which suggests a diminished contribution of attitude changes on intention changes. Similarly, subjective norms were not significantly associated with intentions to take part in regular physical activity. The non-significant relationships between SN and intentions (intercept and slope) changes has been frequently reported in TPB research in other health behaviors [28]. The context in which participants were placed (mandatory physical education) may have diminish the contribution of this variable in the present investigation. For example, mandatory physical education may have diminished the contribution social influences towards participation in physical activity. Further research should put more attention on the social influences that support (or not) adherence to regular physical activity during college.

Significant changes were observed regarding intentions, indicating that after two college years, college students seemed more predisposed to getting involved in PA. This may be explained by the fact that physical education classes may have contributed to raise students’ awareness towards the adoption of regular physical activity. For example, as we described earlier, physical education classes were health-oriented (awareness towards healthy and active lifestyle), and encouraged students to set realistic goals about their physical activity levels. In this regard, analyses of the “changes predict changes” part of the model also revealed interesting results. First, changes in PA were associated with changes in intentions and PBC. Such results are similar to what was reported by antecedent research in multiple health-related behaviors [15, 12]. Secondly, attitude changes were not significantly associated with intention changes. Despite being somewhat contradictory with TPB assumptions, the non-significant relationship between attitude and intentions changes have been demonstrated in antecedent research [29]. As suggested by Druckman and Kam [30], attitudes crystallization towards a behavior is a potential explanation for the non-significant (or less important) contribution of attitudes changes on intention changes. Another explanation may be associated with the observed growth of the participants’ perceived control, which may have reduced the contribution of attitude to change intentions (to a non-significant level).

One of the key findings of the present study is that changes in PBC predicted significantly changes in intentions. As mentioned earlier, such results are concordant with what has been demonstrated by antecedent research [11], which suggests that strong control beliefs could lead to more favorable intentions towards PA. Students’ who feel competent physically and perceived themselves as able to surpass barriers to PA are more inclined to get involved in regular physical activity. However, during this period, perceived control diminished significantly, suggesting that they became more aware of potential obstacles that could reduce their abilities to maintain the behavior. Diminution or stabilization of perceived behavioral control over time is sometimes reported [31], and can be explained by many contextual factors. Due to multiple reasons, perceived control could have evolved over the two college years, and participants, despite have taking part in physical education classes during college, could change their perceptions unfavorably regarding physical activity behaviors. Consequently, students may become more aware about the potential obstacles to regular physical activity, comparatively with their initial perceived behavioral control when they enter college. In that sense, it is crucial for stakeholders to consider perceived control as a key element to develop in further physical education interventions.

In summary, the more students develop positive attitudes towards regular PA, and the more they develop favorable beliefs to overcome barriers to PA (strong attitudes means high perceived control), and their intentions should increase. Such results are particularly interesting for stakeholders who are interested in promoting an active lifestyle during college. In this regard, they should design interventions that will emphasize the enhancement of these constructs. Recently a meta-analysis conducted by Avery and colleagues provided a taxonomy of behavior change techniques for increasing physical activity [32]. Among the most promising approaches, teachers should inform their pupils about the benefits of regular PA, and then contribute to foster favorable attitudes towards physical activity. Goal setting and barrier identification-problem solving should contribute to enhance the students’ perceived behavioral control about performing regular physical activity. They should also provide feedback, and teach learning situations in which students will experience success or achievement in multiple facets of PA, and consequently, enhance their perceived control.

### Sex differences regarding physical activity behaviors

As mentioned earlier, many authors demonstrated sex differences regarding physical activity during college [33]. In this perspective, our results partially support these observations. At the initial level, our analyses revealed gender-associated differences (favoring boys) only in intentions and PA, which is similar with antecedent research. For the other TPB constructs, no other gender differences were observed in LGC analyses, similarly to what was reported by Rhodes [34]. In this regard, our results support the conclusions of Nigg and colleagues [35], who recommend to develop interventions similarly regarding gender. Furthermore, most of the girls’ intercept scores were lower, and consequently, growth parameters tended to be higher for them. From a "changes predict changes" perspective, girls had higher growth trajectories in intentions and physical activity. These significant differences could be explained by the fact that girls had lower initial values for these two constructs, resulting in a higher rate of change over the three college semesters. Such results suggest that researchers should investigate gender-specific trajectories of changes in PA determinants with larger samples. Despite promising insights, this study warrants further research to focus more precisely on gender differences in TPB variables.

### Limitations and future directions

Even if the present study’s design permitted long-term observation of PA correlates, it had some drawbacks. This investigation uniquely considered students who completed their college studies over the 2-year period, that is, a possible selection bias. Motivational aspects, such as responses and involvement in physical education classes could have interfered with patterns of PA, especially in an academic achievement context. Students who withdraw or simply refuse to complete questionnaires may be less motivated towards regular PA. It would have been appropriate to investigate the same outcomes among those who did not complete the study. The present results are representative of students who completed the physical education program, as expected in their respective academic curriculum. We also have to specify that this study was not a TPB-based intervention, but rather an observation of changes in multiple PA correlates. The TPB was the theoretical framework of the study, and could serve for further interventions. In this perspective, additional variables could have been integrated to the TPB model, especially with the college population. Many authors have suggested in the past to integrate some additional determinants to have a better understanding of behavior [36]. As an example, Barfield demonstrated that among college students, exercise identity had a stronger relationship with exercise behaviors, comparatively with the TPB constructs, [37]. In this regard, Ajzen [38] recommend to be cautious when regarding the addition of variables to the TPB. Parsimony of the model (being careful in extending the model) should not be affected, and added variables need to be applicable in most behaviors (compatibility of the theory to all kind of behaviors). Another limitation was sample characteristics. The relatively high proportion of girls (65% of the sample) prevents us from generalizing the results, limiting our interpretation of the gender differences observed. Also, PA measurement could lead to some bias in the estimation of PA. Despite the reliability of subjective PA measures, a direct method of measurement (accelerometer) could have contributed to better estimation of behavior [39].

## Conclusion

This study refined our understanding of the TPB. Future research should focus on a longer term perspective, by measuring TPB variables in multiple time frames (e.g., 3-month post, or 1 year later). Our study also has practical implications for educators who are interested in designing theory-based interventions for behavior change. In this regard, more interventional research should be conducted to clearly identify behavioral change techniques that should be considered in an educational context, such as college-based physical education.

## Supporting Information

S1 File The data file in word excel format (.XLS) (XLS)

S2 File The covariance matrix obtained from the EQS syntax (.PDF) (PDF)
