# Supplementary material for: Analyzing Exercise Behaviors during the College Years: Results from Latent Growth Curve Analysis
Source: PLoS One. 2016 Apr 28;11(4):e0154377. doi: 10.1371/journal.pone.0154377 (PMC4849640; doi:10.1371/journal.pone.0154377)
Supplement: S2 File — (PDF) [file pone.0154377.s002.pdf]

```
EQS, A STRUCTURAL EQUATION PROGRAM          MULTIVARIATE SOFTWARE, INC.
COPYRIGHT BY P.M. BENTLER                   VERSION 6.3 (C) 1985 - 2016 (B113).
```

PROGRAM CONTROL INFORMATION

```
/TITLE
LGC MODELING ARTICLE 3
/SPECIFICATIONS
DATA='C:\Users\lemoyne\Desktop\psy_full_design.ESS';
VARIABLES=503; CASES=195;
METHOD=ML,ROBUST; ANALYSIS=MOMENT; MATRIX=RAW;
MISSING=ML; SE=OBSERVED;
/LABELS

/EQUATIONS
V2  =    *V999 + E2;
V394  =  1F1 + 0F2 + E394;
V395 =   1F1 + 1F2 + E395;
V396 =   1F1 + 2F2 + E396;
V397 =   1F1 + 3F2 + E397;
V398 =   1F1 + 4F2 + E398;
V399 =   1F1 + 5F2 + E399;
V474 =   1F3 + 0F4 + E474;
V475 =   1F3 + 1F4 + E475;
V476 =   1F3 + 2F4 + E476;
V477 =   1F3 + 3F4 + E477;
V478 =   1F3 + 4F4 + E478;
V479 =   1F3 + 5F4 + E479;
V492 =   1F5 + 0F6 + E492;
V493 =   1F5 + 1F6 + E493;
V494 =   1F5 + 2F6 + E494;
V495 =   1F5 + 3F6 + E495;
V496 =   1F5 + 4F6 + E496;
V497 =   1F5 + 5F6 + E497;
V382 =   1F7 + 0F8 + E382;
V383 =   1F7 + 1F8 + E383;
V384 =   1F7 + 2F8 + E384;
V385 =   1F7 + 3F8 + E385;
V386 =   1F7 + 4F8 + E386;
V387 =   1F7 + 5F8 + E387;
V444 =   1F9 + -1F10 + E444;
V445 =   1F9 + 0F10 + E445;
V446 =   1F9 + 1F10 + E446;
V447 =   1F9 + 2F10 + E447;
V448 =   1F9 + 3F10 + E448;
V449 =   1F9 + 4F10 + E449;
F1 =   *V999 + *V2 + D1;
F2 =   *V999 + *V2 + D2;
F3 =   *V999 + *V2 + D3;
F4 =   *V999 + *V2 + D4;
F5 =   *V999 + *V2 + D5;
F6 =   *V999 + *V2 + D6;
F7 =   *V999 + *V2 + *F1 + *F3 + *F5 + D7;
F8 =   *V999 + *V2 + *F2 + *F4 + *F6 + D8;
F9 =   *V999 + *V2 + *F7 + *F5 + D9;
F10 =  *V999 + *V2 + *F8 + *F6 + D10;
```

MAXIMUM LIKELIHOOD ESTIMATES OF MEANS AND COVARIANCE MATRIX
(S) BASED ON THE SATURATED (UNSTRUCTURED) MODEL

VARIABLE	SEXE	INTENT1	INTENT2	INTENT3	INTENT4
	V2	V382	V383	V384	V385
MEAN	1.3179	1.5103	1.2479	1.3962	1.4962

VARIABLE	INTENT5	INTENT6	ATTITU1	ATTITU2	ATTITU3
	V386	V387	V394	V395	V396

MEAN	1.7767	1.6178	3.8380	4.2764	3.8375

VARIABLE	ATTITU4	ATTITU5	ATTITU6	PHYSAC1	PHYSAC2
	V397	V398	V399	V444	V445
MEAN	3.7837	3.8951	4.4877	2.7199	2.7778

VARIABLE	PHYSAC3	PHYSAC4	PHYSAC5	PHYSAC6	SUBJ_NO1
	V446	V447	V448	V449	V474
MEAN	2.6812	2.7378	2.5507	3.6265	5.2935

VARIABLE	SUBJ_NO2	SUBJ_NO3	SUBJ_NO4	SUBJ_NO5	SUBJ_NO6
	V475	V476	V477	V478	V479
MEAN	5.1304	5.1731	5.0163	5.2976	5.1601

VARIABLE	CROY1	CROY2	CROY3	CROY4	CROY5
	V492	V493	V494	V495	V496
MEAN	4.5819	3.9210	3.3543	3.4395	3.8697

VARIABLE	CROY6
	V497
MEAN	2.9432

		SEXE V2	INTENT1 V382	INTENT2 V383	INTENT3 V384	INTENT4 V385
SEXE	V2	0.217				
INTENT1	V382	0.156	2.417			
INTENT2	V383	0.057	1.082	2.382		
INTENT3	V384	0.002	1.219	1.570	2.020	
INTENT4	V385	0.048	1.085	1.203	1.140	1.787
INTENT5	V386	0.043	0.817	0.648	0.814	0.850
INTENT6	V387	0.021	1.240	1.012	0.942	1.089
ATTITU1	V394	-0.005	1.521	1.294	1.150	1.248
ATTITU2	V395	-0.088	1.188	1.362	1.203	1.571
ATTITU3	V396	0.058	1.123	1.296	1.107	1.988
ATTITU4	V397	-0.158	1.552	1.562	1.416	1.982
ATTITU5	V398	0.012	0.948	1.086	0.728	1.774
ATTITU6	V399	-0.024	1.310	1.199	0.808	1.592
PHYSAC1	V444	0.174	1.134	0.816	0.612	0.666
PHYSAC2	V445	0.132	0.955	0.929	0.694	0.758
PHYSAC3	V446	0.090	0.876	1.015	0.794	0.802
PHYSAC4	V447	0.100	0.912	0.988	0.899	1.001
PHYSAC5	V448	0.091	0.880	0.712	0.605	0.925
PHYSAC6	V449	0.055	0.696	0.482	0.392	0.536
SUBJ_NO1	V474	0.024	0.218	0.116	0.148	0.175
SUBJ_NO2	V475	-0.023	0.097	0.224	0.201	0.181
SUBJ_NO3	V476	0.022	0.074	0.409	0.136	0.269
SUBJ_NO4	V477	0.042	0.221	0.176	0.257	0.339
SUBJ_NO5	V478	-0.008	-0.013	0.133	0.072	0.193
SUBJ_NO6	V479	0.006	0.013	0.074	0.226	0.340
CROY1	V492	0.107	1.848	1.615	1.605	1.589
CROY2	V493	0.011	2.100	1.665	1.538	1.626
CROY3	V494	0.283	1.647	1.806	1.623	1.504
CROY4	V495	0.243	1.823	1.674	1.469	2.065
CROY5	V496	0.033	1.495	1.445	1.065	1.624
CROY6	V497	0.091	1.962	1.587	1.220	1.559

		INTENT5 V386	INTENT6 V387	ATTITU1 V394	ATTITU2 V395	ATTITU3 V396
INTENT5	V386	1.417				
INTENT6	V387	0.612	2.362			
ATTITU1	V394	0.689	1.161	5.481		
ATTITU2	V395	0.895	1.453	3.472	7.421	
ATTITU3	V396	1.471	1.285	2.809	4.066	7.650
ATTITU4	V397	1.307	1.778	3.011	3.812	4.695
ATTITU5	V398	1.275	1.105	2.566	3.177	5.115
ATTITU6	V399	1.002	1.872	2.756	4.026	5.101

PHYSAC1	V444	0.513	0.722	1.029	0.852	0.848
PHYSAC2	V445	0.474	0.858	0.912	0.858	0.829
PHYSAC3	V446	0.365	0.769	0.878	0.963	1.162
PHYSAC4	V447	0.490	0.881	0.681	0.836	1.002
PHYSAC5	V448	0.605	0.894	0.641	0.849	1.254
PHYSAC6	V449	0.331	0.637	0.719	0.586	0.738
SUBJ_NO1	V474	0.223	0.141	0.454	0.446	0.520
SUBJ_NO2	V475	0.096	0.265	0.171	0.468	-0.062
SUBJ_NO3	V476	-0.030	0.125	0.190	0.183	0.499
SUBJ_NO4	V477	0.130	0.335	0.289	0.220	0.664
SUBJ_NO5	V478	-0.029	0.121	0.300	0.182	0.361
SUBJ_NO6	V479	0.123	0.175	0.106	0.120	0.449
CROY1	V492	0.907	1.182	3.773	2.554	2.524
CROY2	V493	0.921	1.880	3.266	4.581	3.066
CROY3	V494	0.921	1.650	2.825	3.479	4.530
CROY4	V495	1.050	1.529	3.158	3.974	3.658
CROY5	V496	1.147	2.048	3.119	3.725	3.551
CROY6	V497	1.296	3.056	2.192	3.501	3.548

		ATTITU4 V397	ATTITU5 V398	ATTITU6 V399	PHYSAC1 V444	PHYSAC2 V445
ATTITU4	V397	7.683				
ATTITU5	V398	5.353	8.948			
ATTITU6	V399	4.016	4.103	6.845		
PHYSAC1	V444	1.209	0.779	0.994	1.549	
PHYSAC2	V445	1.083	0.796	1.056	1.003	1.309
PHYSAC3	V446	1.180	0.931	1.098	1.011	0.962
PHYSAC4	V447	0.980	0.596	1.012	0.889	0.976
PHYSAC5	V448	1.122	0.736	1.086	0.857	0.856
PHYSAC6	V449	0.758	0.585	0.873	0.497	0.488
SUBJ_NO1	V474	0.506	0.345	0.277	0.125	0.069
SUBJ_NO2	V475	0.001	0.276	-0.001	-0.043	0.141
SUBJ_NO3	V476	0.253	0.877	0.354	-0.032	0.093
SUBJ_NO4	V477	0.869	0.846	0.396	0.146	0.163
SUBJ_NO5	V478	0.520	0.673	0.290	-0.117	-0.109
SUBJ_NO6	V479	0.395	0.470	0.435	-0.045	0.020
CROY1	V492	3.025	3.026	2.412	1.254	1.001
CROY2	V493	3.309	3.283	3.841	1.316	1.407
CROY3	V494	3.531	2.964	2.828	1.107	0.929
CROY4	V495	4.477	3.923	3.703	1.695	1.514
CROY5	V496	3.887	4.337	3.610	1.319	0.941
CROY6	V497	3.535	2.912	4.984	1.326	1.261

		PHYSAC3 V446	PHYSAC4 V447	PHYSAC5 V448	PHYSAC6 V449	SUBJ_NO1 V474
PHYSAC3	V446	1.431				
PHYSAC4	V447	0.978	1.524			
PHYSAC5	V448	0.896	0.902	1.849		
PHYSAC6	V449	0.518	0.550	0.596	0.803	
SUBJ_NO1	V474	0.118	0.063	0.116	0.120	1.334
SUBJ_NO2	V475	0.251	0.143	0.023	0.120	0.437
SUBJ_NO3	V476	0.211	0.174	-0.018	0.128	0.642
SUBJ_NO4	V477	0.231	0.251	0.114	0.192	0.520
SUBJ_NO5	V478	-0.028	0.002	-0.117	0.157	0.435
SUBJ_NO6	V479	0.073	0.135	-0.004	0.179	0.394
CROY1	V492	1.232	1.000	0.408	0.779	0.562
CROY2	V493	1.365	1.245	1.265	0.966	0.058
CROY3	V494	1.231	0.991	0.740	0.840	0.259
CROY4	V495	1.520	1.786	1.263	1.005	0.275
CROY5	V496	1.381	0.855	0.949	1.046	0.352
CROY6	V497	1.512	1.091	1.456	1.087	0.057

		SUBJ_NO2 V475	SUBJ_NO3 V476	SUBJ_NO4 V477	SUBJ_NO5 V478	SUBJ_NO6 V479
SUBJ_NO2	V475	1.479				

SUBJ_NO3	V476	0.587	1.276			
SUBJ_NO4	V477	0.446	0.796	1.317		
SUBJ_NO5	V478	0.309	0.605	0.433	1.236	
SUBJ_NO6	V479	0.309	0.670	0.764	0.514	1.294
CROY1	V492	-0.239	0.591	0.525	0.557	0.243
CROY2	V493	0.178	0.341	0.556	-0.416	0.227
CROY3	V494	-0.178	0.524	0.642	0.681	0.683
CROY4	V495	0.029	0.430	0.674	0.678	0.657
CROY5	V496	0.323	0.785	0.857	0.880	0.741
CROY6	V497	0.037	0.220	0.565	0.342	0.491

		CROY1 V492	CROY2 V493	CROY3 V494	CROY4 V495	CROY5 V496
CROY1	V492	8.798				
CROY2	V493	5.034	10.362			
CROY3	V494	5.402	5.109	12.544		
CROY4	V495	5.779	6.381	6.597	10.996	
CROY5	V496	4.415	4.946	5.170	5.709	11.147
CROY6	V497	3.863	4.908	6.008	4.961	6.088

		CROY6 V497
CROY6	V497	11.343
